# Predictors for diabetic retinopathy progression—findings from nominal group technique and Evidence review

**DOI:** 10.1136/bmjophth-2020-000579

**Published:** 2020-10-09

**Authors:** Sajjad Haider, Salman Naveed Sadiq, Eniya Lufumpa, Harpreet Sihre, Mohammad Tallouzi, David J Moore, Krishnarajah Nirantharakumar, Malcolm James Price

**Affiliations:** 1 University of Birmingham, Birmingham, UK; 2 Ophthalmology, Royal Victoria Infirmary, Newcastle, UK; 3 Institute of Applied Health Research, University of Birmingham, Birmingham, UK

**Keywords:** public health, retina

## Abstract

**Objectives:**

Risk stratification is needed for patients referred to hospital eye services by Diabetic Eye Screening Programme UK. This requires a set of candidate predictors. The literature contains a large number of predictors. The objective of this research was to arrive at a small set of clinically important predictors for the outcome of the progression of diabetic retinopathy (DR). They need to be evidence based and readily available during the clinical consultation.

**Methods and analysis:**

Initial list of predictors was obtained from a systematic review of prediction models. We sought the clinical expert opinion using a formal qualitative study design. A series of nominal group technique meetings to shorten the list and to rank the predictors for importance by voting were held with National Health Service hospital-based clinicians involved in caring for patients with DR in the UK. We then evaluated the evidence base for the selected predictors by critically appraising the evidence.

**Results:**

The source list was presented at nominal group meetings (n=4), attended by 44 clinicians. Twenty-five predictors from the original list were ranked as important predictors and eight new predictors were proposed. Two additional predictors were retained after evidence check. Of these 35, 21 had robust supporting evidence in the literature condensed into a set of 19 predictors by categorising DR.

**Conclusion:**

We identified a set of 19 clinically meaningful predictors of DR progression that can help stratify higher-risk patients referred to hospital eye services and should be considered in the development of an individual risk stratification model.

**Study design:**

A qualitative study and evidence review.

**Setting:**

Secondary eye care centres in North East, Midlands and South of England.

**Key messages**

**What is already known about this subject?**

- A large list of already identified predictors, for diabetic retinopathy progression was available but had duplications and overlap.

**What are the new findings?**

- With the clinical experts’ opinion using nominal group technique and evidence evaluation we identified a shorter, evidence-based and pragmatic list of 19 predictors, which was also ranked by clinical importance.

**How might these results change the focus of research or clinical practice?**

- This set of candidate predictors can help stratify patients with referable diabetic retinopathy under hospital ophthalmology services through risk stratification.

## Introduction

Diabetic retinopathy (DR) can develop in anyone with diabetes mellitus (DM) and is a major cause of blindness due to damage to, and disruption of the retina (the light-sensitive layer at the back of the eye) leading to loss of sight. DR is a consequence of changes to the blood vessels in this part of the eye leading to leakage of blood and fluid and the formation of abnormal blood vessels.^1^

There has been a global increase in the number of adults with DM from an estimated 108 million in 1980 to 422 million in 2014.^2^ The rise in diabetes prevalence coupled with early detection of DR through better population eye screening coverage has increased the burden of patients with DR to healthcare systems.^3^

In the UK, DR services are organised into the Diabetic Eye Screening Programme (DESP) for lower-risk patients and the hospital eye services (HES) for higher-risk patients with referable DR, with the HES providing treatment, and closer observation of patients. While services may be organised differently in other countries, the care pathways are likely to be similar for these patients. Patients with DR are referred to the HES when they develop clinical signs of the so called sight-threatening retinopathy. The clinical signs based on photographs are the only differentiating features used for this risk stratification. However, approximately 50%–70% of referrals^4 5^ do not need intervention and are observed in the HES for varying periods of time. This is one reason for a demand and capacity mismatch in HES.

There have been successful attempts to optimise the diabetic screening services through risk stratification of patients with the help of a prognostic prediction model.^6^ A similar approach could be used to improve safety and efficiency of DR services in the hospital environment. However, a recent systematic review of prediction models for DR found that none of the 14 models identified were directly applicable for the higher-risk patients in the hospital setting.^7^

Predictors^8^ are at the core of prevention and prediction of a clinical event. Predictors could be, for example, an individual attribute, a clinical feature, a physiological, psychosocial or an environmental factor. Predictor research ranges from ‘exploration’ to ‘confirmation’ to ‘replication’. Exploration refers to a predictor being mentioned in a primary study as a risk factor for being part of the causal pathway. Confirmation is established if a predictor retains prognostic value even after adjustment for other predictors. Replication is the assessment of the predictor in multiple independent studies. Publication and reporting biases are common in predictors’ research.^9^

A prediction model combines two or more predictors (also called prognostic factors) to predict the likelihood of an outcome, for example, DR progression to a treatment requiring stage or loss of vision, before it occurs.^10^ Previous prediction models in the systematic review^7^ have between them used 78 different candidate predictors. However, there are problems with the direct use of these predictors for the HES setting. First, a set of predictors this large is not feasible for use in clinical practice. Second, many of these predictors have not been confirmed. Third, a number of predictors were extrapolated from evidence for macrovascular outcomes like stroke to retinopathy progression inappropriately, for example, ‘smoking’ and ‘ethnicity’.^11^ Fourth, there was significant duplication and overlap between the predictors, for example, diabetic nephropathy and chronic kidney disease. Such highly correlated predictors are unlikely to be independently predictive in the same model.^7^ Finally, there may be predictors for higher-risk patients, not reported in the prediction model studies as they were primarily for low-risk patients.

There were two comprehensive and up to date evidence reviews on the subject of predictors. However, their perspective was quite different from ours. They were a rich resource of evidence and very useful for horizon scanning,^12 13^ but most of the predictors they suggested are still in research domain and not being recorded in the patient notes, so not possible at the moment to be used within prognostic research. We instead used the modelling studies included within the systematic review assuming most of them will be evidence based and for the practical reason that they will have been measured and thus can be extracted from the data.

### Aims

In this qualitative study, we aimed to deduplicate the list of predictors and through clinicians input and evidence assessment to arrive at a shorter list, more clinically relevant to high-risk patients.

### Objectives

1. To identify all the predictors for DR progression in the literature
2. To seek clinicians’ opinion about the most important predictors among them for high-risk patients and to identify any new predictors not yet described.
3. To assess the evidence base from published literature for each predictor identified in objective 2.
4. To finalise a potentially parsimonious set, that is, a minimal number of predictors in a future multivariate model able to give the highest predictive performance.^14 15^

## Methods

### Study design and methods

Our recent systematic review of prognostic modelling studies for the development of DR of any type including maculopathy in patients with diabetes^7^ was the primary source of a list of candidate predictors. The text and the reference lists of the modelling studies were searched for the candidate predictors. A qualitative study design (Delphi) has been used with the aim to prioritise the list of already identified predictors.^16^ Techniques other than nominal group technique (NGT) such as brainstorming, focus group and Delphi methods were considered^17^ along with their technical strengths and limitations. NGT was used to shrink the list of candidate predictors. Primary studies were then evaluated for evidence. In case no reference of the primary study was found in the modelling study, searches were made in Pubmed and Scopus. Subsequently, the evidence behind the shortlisted predictors was evaluated using the Quality in Prognosis Studies (QUIP) tool.^18^ To comply with the PROGRESS (Prognosis research strategy) framework for predictors,^9^ we used a criterion of at least ‘exploration’ (exploring predictor’s relation to prognosis) within primary studies before including them into the list of our candidate predictors for future models, to ensure that the risk of the outcomes of interest can be calculated more precisely by using those predictors by a future model.^10^

#### Nominal group technique

NGT is a qualitative research methodology, where every meeting is a structured small group exercise allowing judgements by individuals to be pooled to arrive at a decision in an uncertain situation.^17 19^ NGT has been used frequently in ophthalmology and medicine^20–28^ and is a highly adaptable method.^29^

An information pack with the predictors list from the systematic review was sent to participating unit before the meeting^7^ (online supplemental appendices A1−A4). Informed consent forms were provided and signed by participants (online supplemental appendix A2). NGT was performed through a series of four meetings, each lasting around 1.5 hours. Meetings were accommodated within clinical governance/audit meetings of four National Health Service (NHS) trusts with the permission of respective research and development directorates. NGT was chosen, because of its modifiable nature allowing brainstorming to decide on importance of predictors, a round-robin for an equal opportunity for all participants, a discussion for clarifications, voting for final decision on ranking and for being time efficient. Each meeting was conducted in four stages (online supplemental appendix A4): from providing background information, round-robin recording of ideas, discussion of the list of ideas and ending in voting for ranking of the ideas generated.

10.1136/bmjophth-2020-000579.supp1 Supplementary data

During the round-robin recording, the facilitator went around the table inviting one item from each participant at a time (to give equal opportunity to all the participants) and recording them on the flip chart. It was left open to participants to choose any number of predictors from the list provided or name their own predictors using their personal experience and insight. We requested participants to restrict their final choices to a total of 15–20 predictors.

The round-robin cycles (within a meeting and between the meetings) were repeated until saturation had been achieved—a point where no new predictors were being added and thus all new information had been obtained.^30 31^ The centres were recruited sequentially and from the second meeting onward, after every meeting cumulative results were examined for any new predictors suggested and thus monitoring for saturation (when no further predictors were being added) before stopping to recruit.

#### Participants

To ensure a maximum range of views and opinions were collected, keeping purposeful sampling in mind that is, selection of information-rich resource for the research question^32^ as a priority, we approached medical retina team leaders for four NHS trusts (consultants with much longer training and experience)^33^ with their teams of middle grades and registrars, nurses and optometrists, directly involved in caring for patients with DR, for these meetings. We aimed to over-recruit allowing for likely no-shows/dropouts on the day for each group.

### Data collection

Reflexivity^34^ of the authors was considered when designing the project. SH is an ophthalmologist and thus shared the participants’ experience. He moderated the meetings, but one or more of the qualitative study researchers (HS, EL or MT) were also present to reduce the chances of bias. Clinicians were asked for their written consent (online supplemental appendix A2) for information collected to be used for further research and publication. Flip chart and marked lists were collected at the end of discussion while voting and all other information was collated on a spreadsheet. All participant data were anonymised, with no direct quotes mentioned in any dissemination.

Data extraction in spreadsheet and collation after every meeting were carried out by one of the coauthors involved in that meeting and validated with the paper forms with the help of a second researcher. We collated all new predictors suggested by clinical colleagues participating in these meetings, added them to the list provided and helped participants rank them for importance in prognosis prediction.

### Analyses of NGT

Voting frequency was calculated for voting scores per predictor (summing of scores) as well as frequency of voting percentage (score achieved for the item/maximum possible score × 100) and tabulated according to their rank.^29^

### Evidence review

In this step, we searched the reference lists of the modelling studies identified in the systematic review^7^ for primary studies that had investigated the predictors selected by NGT and subjected them to critical appraisal using the QUIP tool.^18^ A basic literature search was then performed in Pubmed and Scopus to identify primary studies for predictors where no primary evidence source was referenced in any modelling study. Using the identified information, we summarised the status of each predictor as explored, confirmed or confirmed and replicated in more than one study.

#### Patients and public involvement

While there was no direct patients and public involvement required in this research, we included all the predictors from Individualised Screening for Diabetic Retinopathy (ISDR) model which were chosen by an expert patient panel^35^ to reflect patient input.

#### Ethics

The protocol was evaluated by the four NHS trusts where the NGT was carried out. Since the research was essentially a decision exercise based on clinicians’ expert opinion and did not involve patient data, no ethical approval was required.

## Results

**Figure 1:**
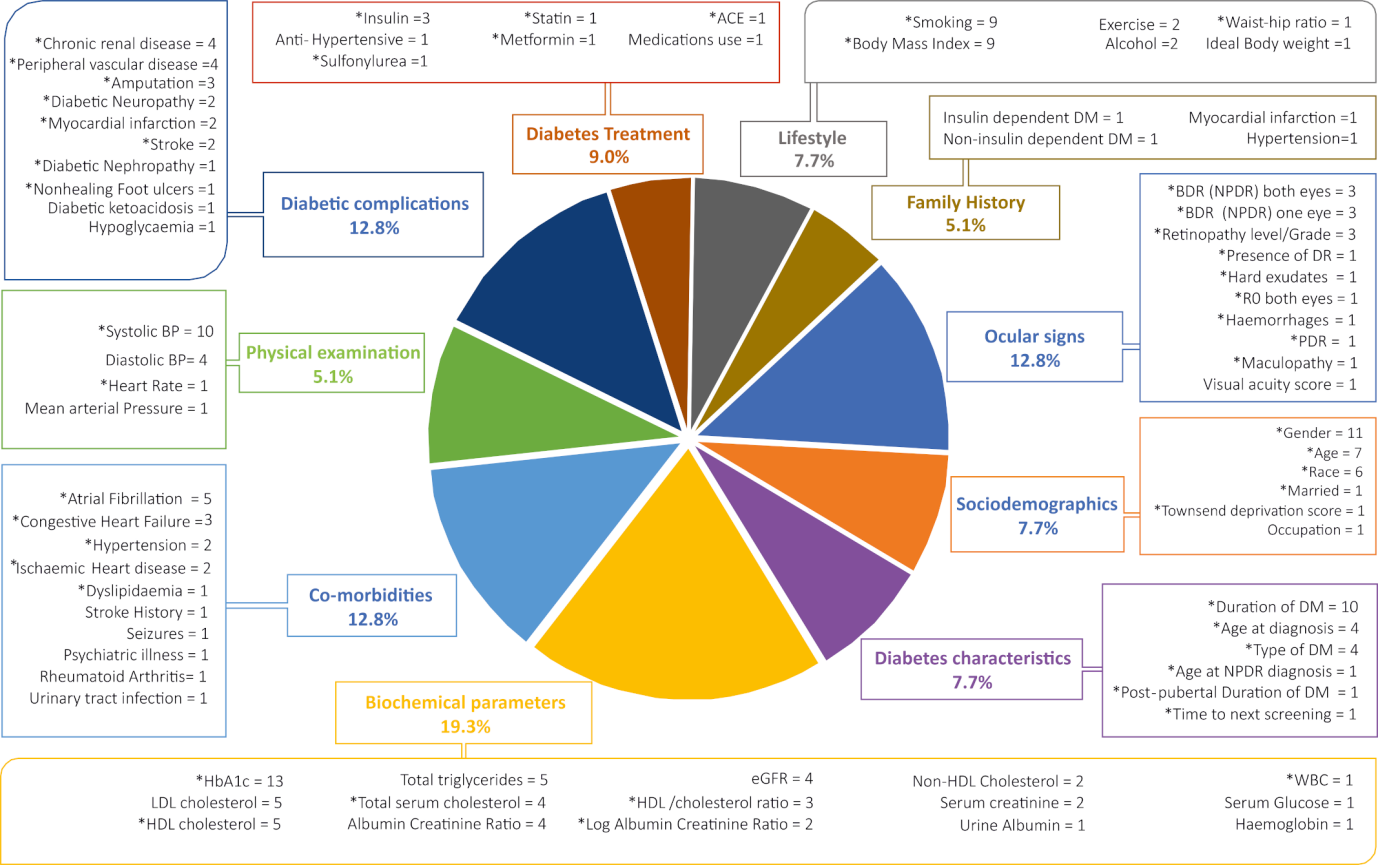
List of candidate predictors from systematic review model development studies. Pie chart illustrates the percentage proportion of each category of predictors. Boxes indicate the type of candidate predictors in each category along with the number of modelling studies that considered them. Information on the full list of candidate predictors is given in online supplemental appendix A3.^7^ *The predictor was used in at least one model development. eGFR, estimated glomerular filtration rate; HbA1c, glycated haemoglobin; HDL, High Density Lipoproteins; LDL, Low Density Lipoproteins; WBC, White Blood Cells; BDR, Background Diabetic Retinopathy; NPDR, Non Proliferative Diabetic Retinopathy; PDR, Proliferative Diabetic Retinopathy.

**Figure 2:**
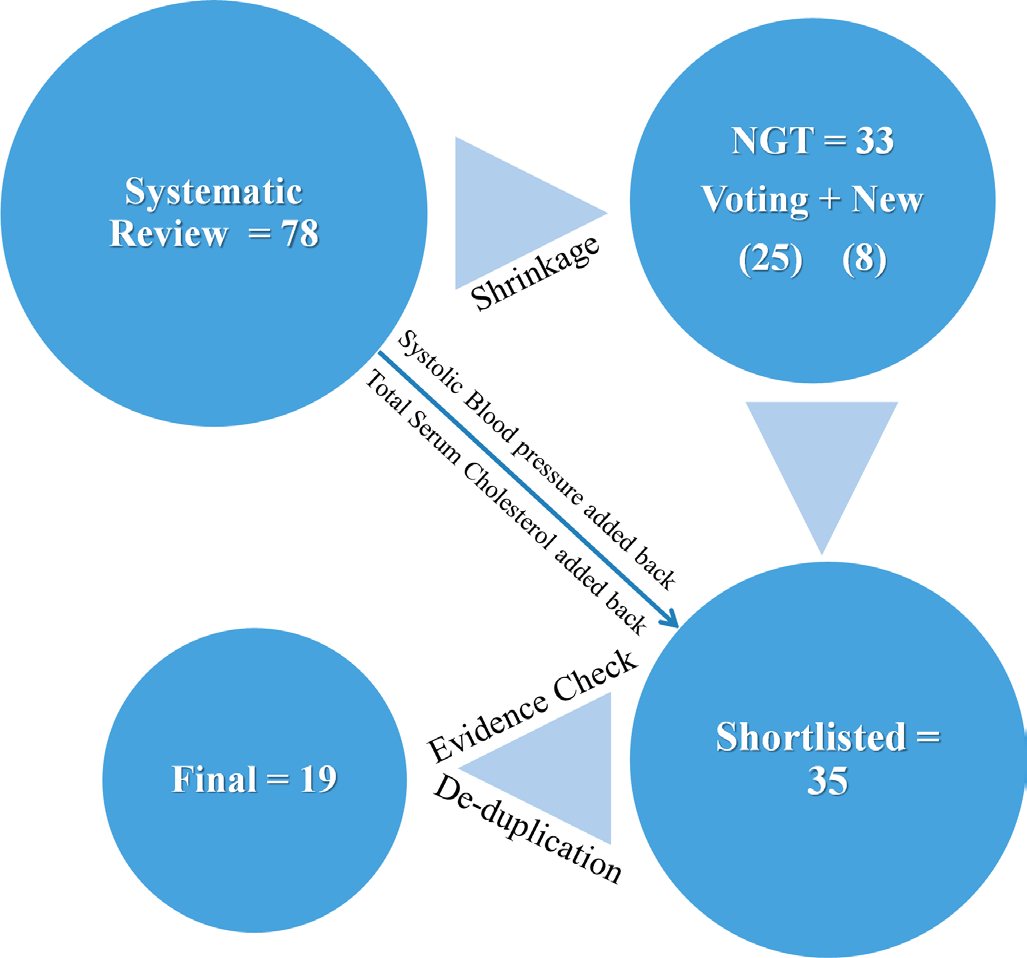
Summary of the sequence involved in reaching the final list of candidate predictors in table 3. NGT, nominal group technique.

The list of 78 candidate predictors from the systematic review are given in figure 1 (shows their breakdown and frequency of their use in the pool of 14 models) and in Appendix A3.^7^ Biochemical predictors were used most commonly. Figure 2 shows the predictor items flow during the processes of NGT and evidence evaluation.

### Nominal group technique

We conducted four NGT meetings, within secondary care eye centres in NHS trusts in the following areas in the UK: (1, 2) Two Midland trusts, (3) North East England and (4) South of England. After the fourth NGT meeting, saturation was reached. The full set of 33 predictors selected by the clinicians are shown in table 1. Participants’ roles varied from consultants, specialist registrars, middle-grade doctors and allied health professionals (one optometrist and one nurse). A total of 44 clinicians participated with numbers per session ranging from 6 to 16 (online supplemental appendix A4). Eleven out of 19 participating consultants were medical retina trained. After multiple round-robin cycles (within a meeting and different meetings) had been repeated to ensure saturation had been achieved and no new predictors were being added (online supplemental appendix A6), the final set of candidate predictors as decided by NGT is given in table 1.

**Table 1:** List of predictors chosen by NGT by number of votes and voting frequency

Predictor		Voting frequency	Proportion voted (total n=44) (%)*
1	HbA1c	31	70
2	Duration of diabetes	24	55
3	Retinopathy level	17	39
4	Townsend score	16	36
5	Smoking (lifestyle)	12	27
6	Race	11	25
7	Proliferative DR	11	25
8	DNA†	10	23
9	Nephropathy	9	20
10	Hypertension	9	20
11	Maculopathy	8	18
12	Pregnancy†	7	16
13	Comorbidities†	7	16
14	Exercise/physical activity	6	14
‘15	Type of DM	5	11
16	BMI	5	11
17	eGFR	4	9
18	Chronic renal disease	4	9
19	Rapid reduction of blood sugar (early worsening)†	4	9
20	Dyslipidaemia	3	7
21	Psychiatric illness	3	7
22	Visual acuity score	2	5
23	DBP	2	5
24	Only eye†	2	5
25	Age at diagnosis	1	2
26	Chronic infection†	1	2
27	Preproliferative†	1	2
28	Neuropathy	1	2
29	Age	1	2
30	Statins	1	2
31	Insulin	1	2
32	Gender	1	2
33	Diet†	1	2

*Voting frequency in percentages arranged in order of high to low. NGT selected 25 out of 78 candidate predictors list.

†Eight new predictors were added by NGT for a total of 33 predictors.

BMI, body mass index; DBP, diastolic blood pressure; DM, diabetes mellitus; DNA, did not attend; DR, diabetic retinopathy; eGFR, estimated glomerular filtration rate; HbA1c, glycated haemoglobin; NGT, nominal group technique.

### Evidence evaluation

Following NGT, during the evidence review, two further predictors from the original list of 78 were added back to give a total of 35 predictors (table 2). ‘Systolic blood pressure’ (SBP) was included because of the participation of patient expert panel in ISDR model,^35^ was the third the most common predictor used in prognostic models and has a good evidence base. The NGT participants probably implicitly included it by selecting hypertension as well. Total serum cholesterol was added back as a proxy for dyslipidaemia as difficulties with reporting of this predictor have previously been reported.^36^

**Table 2:** Predictors and their primary studies with confirmation and replication status

Group	No	Predictors	Model mentioning predictor	Status
Ocular features	1	Retinopathy level/DR grade	Lagani *et al*^14^	C,^48^ R^70^
	2	Proliferative DR	Hippisley-Cox and Coupland^11^*	C,^48^ R^71^
	3	Maculopathy	Hippisley-Cox *et al*^11^*	C,^48^ R^71^
	4	Visual acuity score	Lagani *et al*^14^	C^51^
Sociodemographics	5	Age	Multiple studies^11 42 57^	C,^43^ R^55^
	6	Race	Harris *et al* 11 63 (L)	E^3 63^ Lack of evidence
	7	Gender	Harris *et al*^63^	C,^62^ R^43^
	8	Social deprivation score	Hippisley-Cox and Coupland^11^*	C,^59^ R^72^
Diabetes characteristics	9	Type of DM	Icelandic model^73^*	C^74^
	10	Age at diagnosis	UKPDS OM2^75^*	C,^76^ R^55^
	11	Duration of DM	Icelandic model^73^	C,^48^ R^70^
Biochemical parameters	12	HbA1c	UKPDS OM1^77^	C,^51^ R^49^
	13	eGFR	UKPDS OM2^75^ Stratton *et al*,^60^ ISDR^35^*	C,^78^ R^38^ C,^79^ R^55^
	14	Total serum cholesterol	Soedamah-Muthu *et al*^57^ UKPDS OM1^77^	C,^39^ R^55^ C,^40^ R^53^
Physical examination	15	DBP	Icelandic model^73^*	C^74^
	16	SBP	UKPDS OM2^77^*	C,^76^ R^55^
Comorbidities	17	Hypertension	Harris *et al*^63^	C^39^
	18	Dyslipidaemia	Harris *et al*^63^	C,^79^ R^80^
	29	Psychiatric illness	Lagani *et al*^14^ (L)*	Absence of evidence
Diabetic complications	20	Chronic renal disease	*Hippisley-Cox and Coupland^11^	C^81^
	21	Diabetic nephropathy	Harris *et al*^63^	C,^62^ R^37^
	22	Diabetic neuropathy	Harris *et al*^63^ (L)*	Absence of evidence
Diabetes treatment	23	Statin	Harris *et al*^63^	C,^82^ R^80^
	24	Insulin	Harris *et al*^63^*	C,^83^ R^50^
Lifestyle	25	Smoking	McEwan *et al*^61^ (L)	C,^43 44^ Absence of evidence
	26	BMI	McEwan *et al* and others^14 42 61^ (L)*	C,^41^ Absence of evidence
	27	Exercise/physical activity	Tanaka^13^ (L)	Absence of evidence
New from NGT	28	Only eye situation	NA (L)	Absence of evidence
	29	Early worsening	NA	C,^45^ R^84^
	30	Frequent DNA	NA (L)	C^47^ Lack of evidence
	31	Pregnancy	NA	C,^46^ R^52^
	32	Diet	NA (L)	Absence of evidence
	33	Preproliferative DR	NA	C,^48^ R^85^
	34	Chronic infection	NA (L)	Absence of evidence
	35	Comorbidities	NA (L)	Absence of evidence

*Modelling study did not clearly identify the primary study for the predictor. Details in text.

BMI, body mass index; C, confirmation; DBP, diastolic blood pressure; DM, diabetes mellitus; DNA, did not attend; DR, diabetic retinopathy; E, exploration; eGFR, estimated glomerular filtration rate; HbA1c, glycated haemoglobin; ISDR, Individualised Screening for Diabetic Retinopathy; L, lacking evidence; NA, not applicable; R, replication; SBP, systolic blood pressure; UKPDS, United Kingdom prospective Diabetes Study.

After NGT, residual duplication/overlap still remained, for example, ‘diabetic nephropathy’ and ‘chronic kidney disease’, ‘hypertension’, ‘diastolic blood pressure’ (DBP) and ‘SBP’, ‘dyslipidaemia’, ‘cholesterol’ and ‘statins’. ‘Diabetic nephropathy’ is the primary cause of ‘chronic kidney disease’ characterised by progressive decline of^37^ ‘estimated glomerular filtration rate’ (eGFR). Therefore ‘eGFR’ was retained in preference to ‘chronic kidney disease’ and ‘diabetic nephropathy’ as it is established as a predictor for DR and is more sensitive than the earlier two predictors.^38^ In the case of the overlap between ‘hypertension’, ‘DBP’ and ‘SBP’, the latter two predictors are represented in ‘hypertension’ but seem to have different prognostic values, so were retained.^39 40^

We did not find any primary studies supporting the association between DR progression and ‘psychiatric illness’ or ‘diabetic neuropathy’. Among the studies found, no proven association of DR with ‘BMI’,^41^ ‘exercise/physical activity’^42^ or ‘smoking’^43 44^ was seen but a weak association was seen with ‘serum cholesterol’. The last item was therefore retained, and the rest excluded.

Among the new predictors suggested by NGT, primary studies were found confirming the association for ‘early worsening’,^45^ ‘pregnancy’,^46^ frequent ‘DNA’,^47^ and ‘preproliferative DR’.^48^ Due to the lack of evidence, the remaining four predictors (‘comorbidities’, ‘only eye situation’, ‘Diet’’ and ‘chronic infection’) were excluded.

The most common primary studies quoted were UKPDS,^39 40 43 48–50^ Diabetes Control and Complications Trial (DCCT),^45 51 52^ Wisconsin Epidemiological Study of Diabetic Retinopathy,^53 54^ Epidemiology and Prevention of Diabetes Study (EURODIAB)^55^ and Action to control cardiovascular risk in diabetes (ACCORD).^56^ Table 2 tabulates primary studies and status of confirmation and replication for individual predictors. Fourteen predictors were excluded for reasons of no proven association, duplication/overlap. The remaining 21 predictors were condensed into 19 predictors (table 3) by combining the DR categories together.

**Table 3:** Final list of candidate predictors

	Group	Predictors
1	Ocular features	Presence and DR grade Proliferative DR Preproliferative DR* Maculopathy
2		Visual acuity score
3	Sociodemographic	Age at STR diagnosis
4		Age
5		Race
6		Gender
7		Social deprivation score
8	Diabetes characteristics	Type of DM
9		Duration of DM >10 years
10	Biochemical parameters	HbA1c
11		eGFR
12		Total serum cholesterol
13	Physical examination	SBP
14		DBP
15	Diabetes treatment	Statin
16		Insulin
17	NGT*	Pregnancy
18		Early worsening
19		Frequent DNA

*Preproliferative DR, new from NGT is moved up to appear with the rest of DR categories, 4 condensed into 1, and thus 21 predictors condensed into 19. Also ‘age at diagnosis’ was specified to ‘age at STR diagnosis’ to conform with our target population of referable DR.

DBP, diastolic blood pressure; DM, diabetes mellitus; DR, diabetic retinopathy; eGFR, estimated glomerular filtration rate; HbA1c, glycated haemoglobin; NGT, nominal group technique; SBP, systolic blood pressure; STR, sight-threatening retinopathy.

Only 2 out of 14 modelling studies^42 57^ reported primary studies for all of the prognostic factors/predictors used. One of the modelling studies used the items without giving reference to any primary study^35^ but instead quoted literature review/expert patients’ panel. A model partly relied on borrowing the items from other models,^42^ some could be traced from the reference list but were not in context.^42 58^ It was not clear in 8 (23%) out of 35 predictors, as to which primary studies confirmed them (given in table 2).

Most of the primary studies had multiple publications. Out of 35 predictors, 25 (71%) had good supporting evidence of predictive value from the literature (table 2). Four predictors were excluded because of overlap/duplication. Out of 18 primary studies critically appraised in online supplemental appendix A7, the evidence base for only one primary study had a high risk of bias, mainly due to confounders issues and highly selective population.^59^ The factor involved did have another supporting study with low risk of bias. Three predictor studies were judged to have moderate risk of bias and 15 predictor studies were low risk of bias (79% of primary predictors studies) on QUIP criteria.

## Discussion

### Statement of principal findings

We used clinical opinion in the NGT meetings to reduce a list of 78 previous candidate predictors to a list of 25 potential predictors. The study also suggested a further eight potential predictors (table 1). After evidence review, we added back another two predictors.

The new predictors suggested by the NGT made good clinical/biological sense, but four of them (‘Comorbidities’, ‘only eye situation’, ‘diet’, ‘chronic infections’) have not been explored for association with DR progression, although it is possible that the last two may be operational through other variables such as glycated haemoglobin (HbA1c) rise due to uncontrolled blood sugar. The ‘only eye’ situation clearly has a higher risk of blindness because of the absence of function in the affected eye, or higher risk because of the same pathology as the lost eye but has not been investigated for its association with DR progression. The other three predictors, ‘early worsening’, ‘pregnancy’ and ‘preproliferative DR’ have a proven association with our outcome of interest and were therefore included. While specific comorbidities were considered as mentioned above, ‘comorbidities’ presence or numbers as a predictor had no supporting evidence. ‘Frequent DNA’ has been proven to add prognostic value, so has been added to the final list but is in need for further confirmation.

The top three ranked predictors from NGT (HbA1c, duration of diabetes and DR grade) were the same as the low risk of bias models’ predictor sets (online supplemental appendix A7) chosen by the systematic review.^7^ This was in spite of this information being withheld from the participants in the NGT meetings. This shows the clinicians’ intuitive thinking matches the findings of the systematic review as well. The approach of using retinopathy stages data alone to develop a risk stratification tool in DESP environment has been suggested.^60^ ISDR model^35^ in complete contrast has suggested using clinical predictors alone, especially for higher-risk patients. We looked at risk estimates of the various predictors within various models. They tend to vary depending on the combination of predictors used.^6 14^ However, ocular predictors generally showed higher relative risk estimates. We suggest that any future model should contain a combination of ocular and systemic predictors for use in higher risk hospital population, as the values of, for example, SBP and HbA1c and so on are likely to be higher and can thus add significantly to the predictive ability. A practical sized set is now available, with individual predictors ranked in importance as perceived by the NGT participants. A prediction model for higher-risk DR could be built based on them in appropriate population data.

Several predictors in previously reported models were associated with diabetic complications other than retinopathy—in multiple outcomes or composite outcome models.^14 57^ The evidence for association of some of these predictors with DR is unclear. For example, DR was a main covariate for severity of ‘diabetic polyneuropathy’,^14 39^ but it still remains to be seen if the reverse is also true. ‘Smoking’ as a candidate predictor was included in the majority of the models but made it to the final set only in one of them.^61^ The primary studies failed to show it as a predictor for DR progression.^43 62^ Harris *et al*^63^ included ethnicity in their final model but HR p value crossed the recommended threshold of >0.2, so was of doubtful statistical significance. It was included in five models as candidate predictor but not made to any of the final set on statistical testing. However, there is indirect evidence of higher risk of south Asians to develop STR^3^ and blacks and South Asians among patients under DESP having a higher prevalence of visual impairment.^64^ We therefore included ethnicity in the final set.

### Strengths and weaknesses of the study

We based this study on the full list of predictors that had duplication, overlap and extrapolation of evidence from outcomes other than DR progression. In this situation with a large number of predictors already confirmed and replicated, incorporating clinical insight through a qualitative study design was invaluable to reach a shorter, more manageable list and also generate some new predictors. This is also the first study to evaluate the evidence base of potential predictors used in existing prognostic models for DR progression and to follow the Prognosis Research strategy (PROGRESS) framework recommendations.^9^ We noted reporting deficiencies and have suggested possible preventive strategies for future.

### Strengths and weaknesses in relation to other studies

The ISDR model^35^ mentioned using a patient expert panel for decision-making during the predictor selection process. There is not enough detail in the study design, and we assume that it is only comparable to our work in that the study design was a qualitative one. We provide here the details of our methods and results with the interpretation. Our list also includes all the ISDR predictors, reflecting their expert patients’ input. The Standardisation of Uveitis Nomenclature^20^ and Consensus on Outcome Measures for Glaucoma Effectiveness Trials^25^ are prime examples of successful use of NGT to arrive at a decision with the help of expert panels in ophthalmology. Another qualitative study looked into the patient-perceived risks of disease and benefits of treatment^65^ but did not address predictors selection. We wanted to build on this approach seeking clinical experts’ opinion with the help of NGT meetings.

### Implications for clinicians and policymakers

This set of predictors derived will be useful to risk stratify patients, optimise treatment strategies, inform patients about their personal risks and improve research strategies as well as providing the building blocks for future prognostic models.^9^ A set of predictors based on these 19 finalised predictors could be used to risk stratify the population received within the HES after referral from DESP, to help with prioritisation of appointments and thus direct the resources more appropriately. Alternatively, a model could be constructed to estimate an individual patient’s risk. This research will help the clinicians manage their patients according to their risk of progression.

While we have attempted to develop a list of predictors that are useful in predicting patients who progress from referable DR to a stage of needing treatment or vision loss, the list is primarily from patients with diabetes under screening for incident DR and referable DR and as such are generalizable as markers for progression to any stages.

### Unanswered questions and future research

Risk of bias assessment did not affect our decision to exclude any predictors as vast majority of the predictors had good evidence base. PROGRESS criteria was used to decide which predictors were not confirmed yet. That has helped decide where there is need for further research, for example, ‘Race’, ‘diet’, ‘exercise’ and so on.

We also found the reporting of the evidence base for the predictors selected in the modelling studies suboptimal. Under-reporting has been mentioned by other observers before^9^ and needs improving. The Transparent Reporting of a multivariable prediction model for Individual Prognosis or Diagnosis (TRIPOD) statement asks for all predictors used to be clearly defined.^66^ One way of improving this situation may be for TRIPOD checklist to encourage investigators to report the primary study of origin for every candidate predictor used in a model. Out of 35, 10 (29%) predictors were not supported by the existing evidence base applying PROGRESS standards and require further research.

There have been recent useful reviews and studies on ocular predictors and their natural history use as predictors of DR progression^5 12 13 67^ Ocular signs identified in Optical coherence tomography and Fundus Fluorescein Angiography could also be potential predictors of DR progression. While the ocular signs recommended are suitable for prospective research studies, existing retrospective longitudinal data most commonly used for prognostic research are unlikely to have sufficient information on these predictors. There is an ever-increasing interest in fundus images-based detection, assisted by artificial intelligence.^68^ Prognostic factor research is a dynamic field and will benefit greatly with these newer technologies. Machine learning can handle the data from wider sources, can bring additional benefits from automation, unsupervised clustering of a much larger number of predictors and can also add new phenotypes associated with the outcomes. However, there are ethical, governance, interpretability issues and the process of development of these techniques are at an early stage of development and application.^69^ It is likely that future research will identify further potentially important predictors so an update may be required in the future.

## Conclusion

We have been able to identify 19 evidence-based predictors for DR progression, using a novel method (NGT) and evidence review inline with PROGRESS recommendations. This smaller and more practical set provides a useful resource for a potential model to stratify patients for risk of DR progression, to aid clinical decision-making and optimise clinical care pathways. This set is ranked in importance by the NGT.
