# Supplementary material for: Predictors for diabetic retinopathy progression—findings from nominal group technique and Evidence review
Source: BMJ Open Ophthalmol. 2020 Oct 9;5(1):e000579. doi: 10.1136/bmjophth-2020-000579 (PMC7549478; doi:10.1136/bmjophth-2020-000579)
Supplement: Supplementary data [file bmjophth-2020-000579supp001.pdf]

Appendix A1: Participant information sheet

Prognostic Prediction Factors for Diabetic Retinopathy (DR) progression

These patients are referred to the hospital eye services (HES) when they develop clinical signs of sight-threatening diabetic retinopathy. However, approximately 50% of referrals don't need intervention and are observed in the HES for a variable period of time.

- As clinicians, you may have felt the necessity for a risk stratification tool supporting your decision in; 1) which patients to prioritise for more urgent appointments 2) whether to definitely be able to see them before they reach treatment stage or vision loss and need evidence to support your decision. Such a tool will need certain candidate predictors and we have put together a set (Appendix A3) from our systematic review of published tools /models (mainly for screening patients).
- This study is a step to develop a prediction model for the progression of diabetic retinopathy with an aim to help predict the above outcomes safely and effectively.
- *Please help us select top prognostic factors which can be factors on this list or any additional factors that you can think of but is not on this list.*
- *To do that you may wish to write down without looking at the list given, ignore the order these factors are listed in or the groups they belong to and decide on the basis of your own personal knowledge and experience.*

Thanks for agreeing to take part in this study. Please mark on the list in Appendix A3. Please feel free to add at the end of the list any additional factors, name and sign the consent form and return at the end of the meeting.

Appendix A2: Participant consent form

	Please write your initials in the Box
1. I confirm that I have read and understand the information sheet dated 31/08/2018 (version 1) for the above study. I have had the opportunity to consider the information ask questions and have had these answered satisfactorily.	☐
2. I understand that my participation is voluntary and that I am free to withdraw at any time without giving any reason.	☐
3. I understand that relevant sections of my notes and data collected during the study may be looked at by individuals above, from regulatory authorities or from the NHS Trust, where it is relevant to my taking part in this research. I give permission for these individuals to have access to my input.	☐
4. I consent to this material being used for research and publications.	☐
5. I freely agree to take part in this study	☐

Name of Participant	Date	Signature
Name of Researcher	Date	Signature

Appendix A3: List of candidate predictors from the Systematic Review

**PREDICTOR GROUPS (n = type of candidate predictors in each group)**	
Ocular signs (10)	Retinopathy level<br>Presence of DR<br>Hard exudates<br>R0 both eyes<br>BDR (NPDR) both eyes<br>BDR (NPDR) one eye<br>Haemorrhages<br>PDR<br>Maculopathy<br>Visual acuity score
Socio-demographics (6)	Age<br>Gender<br>Race<br>Married<br>Townsend deprivation score<br>Occupation
Diabetes characteristics (6)	Type of DM<br>Age at diagnosis<br>Age at NPDR diagnosis<br>Duration of DM<br>Post-pubertal Duration of DM<br>Time to next screening
Biochemical parameters (15)	HbA1c<br>Serum Glucose<br>HDL /cholesterol ratio<br>Total serum cholesterol<br>LDL cholesterol<br>HDL cholesterol<br>Non-HDL Cholesterol<br>Albumin /creatinine ratio<br>Urine Albumin<br>Serum creatinine<br>Total triglycerides<br>Log ACR

	Haemoglobin<br>eGFR<br>WBC
Physical examination (4)	SBP<br>DBP<br>Heart Rate<br>Mean arterial pressure
Co-morbidities (10)	HTN<br>Stroke History<br>CHF history<br>IHD history<br>Dyslipidaemia<br>Atrial Fibrillation<br>Seizures<br>Psychiatric illness<br>Rheumatoid Arthritis<br>Urinary tract infection
Diabetic complications (10)	Chronic renal disease<br>Nephropathy<br>Non-healing foot ulcer<br>Amputation<br>Stroke<br>Neuropathy<br>Peripheral vascular disease<br>MI<br>Diabetic ketoacidosis<br>Severe Hypoglycaemia
Diabetes treatment (7)	Statin<br>Metformin

	Sulfonylurea<br>Insulin<br>ACE<br>Anti-Hypertensive Rx<br>Medications use
Lifestyle (6)	Smoking<br>Ideal Body weight<br>BMI<br>Exercise/physical activity<br>Alcohol<br>Waist-hip ratio
Family History (4)	IDDM<br>NIDDM<br>MI<br>HTN
**Total candidate predictors = 78**	

* Compiled from Haider S, Sadiq SN, Moore D, Price MJ, Nirantharakumar K. Prognostic prediction models for diabetic retinopathy progression: a systematic review. Eye. 2019;33(5):702-13

Appendix A4: The four-stage NGT Process (1)

**1**	Silent generation of ideas in writing (Brainstorming):	At the beginning of the meeting, participants were given an information sheet (Appendices A1 to A4) which includes a brief overview of the study, and the list of prognostic factors to select from and add to.	This was sent to participants before the meeting as well
**2**	Round-robin recording of ideas	Each participant was asked to propose one item that they believed is the most important predictor for DR progression. This happened over several rounds until there were no new predictors proposed.	Participants were asked to keep it simple and precise - One phrase only. Each prognostic factor mentioned was written on a flipchart.
**3**	Discussion of the list of ideas	Participants were encouraged to discuss their generated list of predictors and ask for any clarifications needed	An equal opportunity was provided for all
**4**	Voting	Using five stickers, each participant was asked to place stickers next to their top five predictors written on the flip chart from step 2.	Retina specialists were given a different coloured sticker, to be able to analyse two groups separately if needed

Appendix A5: Breakdown of participants

Grades	Midlands NHS Trust	North East NHS Trust	South of England NHS Trust	Midlands NHS Trust	Total
Medical Retina	2	1	4	4	11
Other Consultants	3	4	1	0	8
Associate specialists	1	3	0	1	5
Speciality Doctors	2	3	1	0	6
Registrars	7	1	3	1	12
Nurses	1	0	0	0	1
Optometrists	0	1	0	0	1
Total	16	13	9	6	44

Appendix A6: New factors introduced in the NGT meetings and saturation[a]

	Midlands	North East	South England	Midlands
Only eye situation	Y	-	-	-
Early Worsening	Y	-	-	-
Frequent DNA	-	Y	-	-
Pregnancy	-	Y	Y	Y
Diet	-	Y	-	-
Pre proliferative DR	-	-	Y	Y
Chronic infection	-	-	Y	-
Co-morbidities	Y	-	-	Y

[a] Please see

Appendix A7: Primary studies Risk of Bias

STUDY	Reference Number	RISK OF BIAS (LOW, MODERATE, HIGH)
**Non - Systematic Review**		
Gupta et al	(1)	Moderate
UKPDS 50	(2)	Low
UKPDS 52	(3)	Low
DCCT	(4)	Low
Yau JW et al (Global)	(5)	Moderate
Lane et al	(6)	High
Denniston et al	(7)	Low
Ohkubo Y et al	(8)	Low
Grausland et al	(9)	Low
Jeng et al	(10)	Moderate
Zhou Y et al	(11)	Low
Foster et al	(12)	Low
**Systematic Review**		
WESDR	(13)	Low
DCCT	(4)	Low
ACCORD	(14)	Low
EURODIAB	(15)	Low
UKPDS 68	(16)	Low
Mehlson et al	(17)	Low
Lasgani et al	(18)	Low

.

References:

1. Gupta A, Gupta V, Thapar S, Bhansali A. Lipid-lowering drug atorvastatin as an adjunct in the management of diabetic macular edema. Am J Ophthalmol. 2004;137(4):675-82.
2. Stratton IM, Kohner EM, Aldington SJ, Turner RC, Holman RR, Manley SE, et al. UKPDS 50: risk factors for incidence and progression of retinopathy in Type II diabetes over 6 years from diagnosis. Diabetologia. 2001;44(2):156-63.
3. Kohner EM, Stratton IM, Aldington SJ, Holman RR, Matthews DR, Group UKPDS. Relationship between the severity of retinopathy and progression to photocoagulation in patients with Type 2 diabetes mellitus in the UKPDS (UKPDS 52). Diabetic Medicine. 2001;18(3):178-84.
4. DCCT. The effect of intensive diabetes treatment on the progression of diabetic retinopathy in insulin-dependent diabetes mellitus. The Diabetes Control and Complications Trial. Archives of ophthalmology (Chicago, Ill : 1960). 1995;113(1):36-51.
5. Yau JW, Rogers SL, Kawasaki R, Lamoureux EL, Kowalski JW, Bek T, et al. Global prevalence and major risk factors of diabetic retinopathy. Diabetes care. 2012;35(3):556-64.
6. Lane M, Mathewson PA, Sharma HE, Palmer H, Shah P, Nightingale P, et al. Social deprivation as a risk factor for late presentation of proliferative diabetic retinopathy. Clinical ophthalmology (Auckland, NZ). 2015;9:347-52.
7. Ohkubo Y, Kishikawa H, Araki E, Miyata T, Isami S, Motoyoshi S, et al. Intensive insulin therapy prevents the progression of diabetic microvascular complications in Japanese patients with non-insulin-dependent diabetes mellitus: a randomized prospective 6-year study. Diabetes research and clinical practice. 1995;28(2):103-17.
8. Grauslund J, Green A, Sjolie AK. Blindness in a 25-year follow-up of a population-based cohort of Danish type 1 diabetic patients. Ophthalmology. 2009;116(11):2170-4.
9. Jeng CJ, Hsieh YT, Yang CM, Yang CH, Lin CL, Wang IJ. Diabetic Retinopathy in Patients with Diabetic Nephropathy: Development and Progression. PloS one. 2016;11(8):e0161897.
10. Zhou Y, Zhang Y, Shi K, Wang C. Body mass index and risk of diabetic retinopathy: A meta-analysis and systematic review. Medicine (Baltimore). 2017;96(22):e6754.
11. Klein R, Klein BE, Moss SE, Davis MD, DeMets DL. The Wisconsin epidemiologic study of diabetic retinopathy. IV. Diabetic macular edema. Ophthalmology. 1984;91(12):1464-74.
12. Accord Study Group, Accord Eye Study Group, Chew EY, Ambrosius WT, Davis MD, Danis RP, et al. Effects of medical therapies on retinopathy progression in type 2 diabetes.[Erratum appears in N Engl J Med. 2011 Jan 13;364(2):190], [Erratum appears in N Engl J Med. 2012 Dec 20;367(25):2458]. New England Journal of Medicine. 2010;363(3):233-44.
13. Sjolie AK, Stephenson J, Aldington S, Kohner E, Janka H, Stevens L, et al. Retinopathy and vision loss in insulin-dependent diabetes in Europe. The EURODIAB IDDM Complications Study. Ophthalmology. 1997;104(2):252-60.
14. Clarke PM, Gray AM, Briggs A, Farmer AJ, Fenn P, Stevens RJ, et al. A model to estimate the lifetime health outcomes of patients with type 2 diabetes: the United Kingdom Prospective Diabetes Study (UKPDS) Outcomes Model (UKPDS no. 68). Diabetologia. 2004;47(10):1747-59.
15. Mehlsen J, Erlandsen M, Poulsen PL, Bek T. Identification of independent risk factors for the development of diabetic retinopathy requiring treatment. Acta Ophthalmol. 2011;89(6):515-21.
16. Lagani V, Chiarugi F, Thomson S, Fursse J, Lakasing E, Jones RW, et al. Development and validation of risk assessment models for diabetes-related complications based on the DCCT/EDIC data. Journal of diabetes and its complications. 2015;29(4):479-87.

Appendix A8: Predictors distribution in models with low to moderate risk of bias and low concern of applicability (1)

	DR grade	Age	Gender	Age at diagnosis	Duration of diabetes	Type of diabetes	HBA1c	Total serum cholesterol	SBP
Aspelund T et al. 2011(2)	Y	-	Y	-	Y	Y	Y	-	Y
Scanlon PH 2015 (3)	Y	-	-	-	Y	-	Y	-	-
ISDR model 2017 (4)	-	-	-	Y	Y	-	Y	Y	Y
7 common ones among 14 models	Y	Y	Y	Y	Y	-	Y	-	Y

References:

1. Haider S, Sadiq SN, Moore D, Price MJ, Nirantharakumar K. Prognostic prediction models for diabetic retinopathy progression: a systematic review. Eye. 2019.
2. Aspelund T, Porisdottir O, Olafsdottir E, Gudmundsdottir A, Einarsdottir AB, Mehlsen J, et al. Individual risk assessment and information technology to optimise screening frequency for diabetic retinopathy. Diabetologia. 2011;54(10):2525-32.
3. Scanlon PH, Aldington SJ, Leal J, Luengo-Fernandez R, Oke J, Sivaprasad S, et al. Development of a cost-effectiveness model for optimisation of the screening interval in diabetic retinopathy screening. Health Technol Assess. 2015;19(74):1-116.
4. Eleuteri A, Fisher AC, Broadbent DM, Garcia-Finana M, Cheyne CP, Wang A, et al. Individualised variable-interval risk-based screening for sight-threatening diabetic retinopathy: the Liverpool Risk Calculation Engine. Diabetologia. 2017.
